# *In situ* Prokaryotic and Eukaryotic Communities on Microplastic Particles in a Small Headwater Stream in Germany

**DOI:** 10.3389/fmicb.2021.660024

**Published:** 2021-11-29

**Authors:** Alfons R. Weig, Martin G. J. Löder, Anja F. R. M. Ramsperger, Christian Laforsch

**Affiliations:** ^1^Genomics and Bioinformatics, Bayreuth Center of Ecology and Environmental Research, University of Bayreuth, Bayreuth, Germany; ^2^Animal Ecology, Bayreuth Center of Ecology and Environmental Research, University of Bayreuth, Bayreuth, Germany; ^3^Biological Physics, University of Bayreuth, Bayreuth, Germany

**Keywords:** biofilm, prokaryote community, eukaryote community, freshwater stream, microplastics

## Abstract

The ubiquitous use of plastic products in our daily life is often accompanied by improper disposal. The first interactions of plastics with organisms in the environment occur by overgrowth or biofilm formation on the particle surface, which can facilitate the ingestion by animals. In order to elucidate the colonization of plastic particles by prokaryotic and eukaryotic microorganisms *in situ*, we investigated microbial communities in biofilms on four different polymer types and on mineral particles in a small headwater stream 500 m downstream of a wastewater treatment plant in Germany. Microplastic and mineral particles were exposed to the free-flowing water for 4 weeks in spring and in summer. The microbial composition of the developing biofilm was analyzed by 16S and 18S amplicon sequencing. Despite the expected seasonal differences in the microbial composition of pro- and eukaryotic communities, we repeatedly observed polymer type-specific differentiation in both seasons. The order of polymer type-specific prokaryotic and eukaryotic community distances calculated by Robust Aitchison principal component analysis (PCA) was the same in spring and summer samples. However, the magnitude of the distance differed considerably between polymer types. Prokaryotic communities on polyethylene particles exhibited the most considerable difference to other particles in summer, while eukaryotic communities on polypropylene particles showed the most considerable difference to other spring samples. The most contributing bacterial taxa to the polyethylene-specific differentiation belong to the Planctomycetales, Saccharimonadales, Bryobacterales, uncultured Acidiomicrobia, and Gemmatimonadales. The most remarkable differences in eukaryotic microorganism abundances could be observed in several distinct groups of Ciliophora (ciliates) and Chlorophytes (green algae). Prediction of community functions from taxonomic abundances revealed differences between spring and summer, and – to a lesser extent – also between polymer types and mineral surfaces. Our results show that different microplastic particles were colonized by different biofilm communities. These findings may be used for advanced experimental designs to investigate the role of microorganisms on the fate of microplastic particles in freshwater ecosystems.

## Introduction

Plastic materials are widely used in all areas of human life due to their outstanding material properties such as lightweight, stability, corrosion resistance, insulating properties, and moldability. Global plastic production increased from 1.5 million tons in 1950 to 367 million tons in 2020 (Statistica, 2021). The primary uses for plastics in Europe are packaging (39.6%), followed by building and construction, automotive, electrical/electronic, household/leisure/sports, and agriculture (PlasticsEurope, 2020). The polymer types used for the production of packaging materials are mainly polyethylene (PE; in different densities), polypropylene (PP), and polyethylene terephthalate (PET), and to a lesser extent polystyrene (PS) and polyvinylchloride (PVC). The often short-time use of plastics in packaging contributes mainly to the enormous increase of plastic waste. Of the plastic waste that was collected appropriately in Europe in 2018, only 32.5% entered the recycling process, while the rest of the collected waste was used for energy recovery by incineration (42.6%) or disposed in landfills (24.9%) (PlasticsEurope, 2020). However, it is estimated that 33% of global waste is not collected appropriately but openly dumped or littered, imposing unforeseen risks to public and environmental health (Kaza et al., 2018).

Once plastic material enters the environment, it fragments into smaller particles (microplastics smaller than 5 mm) (Arthur et al., 2009). The fragmentation and eventually degradation processes depend on various physicochemical factors such as mechanical forces, temperature, UV radiation, pH, and additives present in the plastic and varies between different polymer types (Barnes et al., 2009). In addition, plastic degradation by environmental (micro-)organisms can add to the overall degradation process (Restrepo-Montoya et al., 2011), a fact summarized by Yuan et al. (2020) in a recent review. Therefore, the direct interaction of microorganisms with plastics could be of key importance for the fate of plastic in the environment, as it has been shown for PET-and polyurethane (PU)-degrading bacteria (Nakajima-Kambe et al., 1999; Yoshida et al., 2016) as well as for PU- and PE-degrading fungi (Brunner et al., 2018; Sangale et al., 2019).

The composition of biofilms on plastic particles in natural environments, the so-called “plastisphere” (Zettler et al., 2013), has been investigated in aquatic (e.g., marine or freshwater) and terrestrial environments (for reviews, see Oberbeckmann et al., 2015; Oberbeckmann and Labrenz, 2020). Recently, a thorough meta-analysis of studies describing the global diversity of the plastisphere published between 2010 and 2019 revealed the large variety of experiments (Wright et al., 2021): of the 35 studies selected for the meta-analysis, only 6 studies included field studies in the water column of freshwater habitats (Hoellein et al., 2014, 2017; McCormick et al., 2014, 2016; Arias-Andres et al., 2018; Parrish and Fahrenfeld, 2019), sometimes combined with laboratory experiments and/or exposition experiments in sediments. However, reports on plastisphere biofilms from small headwater streams (in contrast to rivers and lakes) are rare (Kaevska et al., 2016), even though those seemingly pristine aquatic habitats receive plastic particles, e.g., *via* runoff (Piehl et al., 2018), aerial deposition (Zhang et al., 2020), or discharge of sewage effluents. Moreover, in addition to compositional and functional analyses of plastisphere biofilms, the spatial structure of microorganisms has been investigated by advanced confocal microscopy and illustrated analytical access to biofilm development at micrometer scales (Schlundt et al., 2020).

Biofilms are composed of microorganisms, mainly bacteria, although fungi and other micro-eukaryotes can contribute to and be enclosed in biofilms, bound in a mucilaginous matrix of an extracellular polymeric material such as exopolysaccharides (Flemming and Wingender, 2010). Biofilms represent key sites for enzymatic activity, organic matter cycling, respiration, and primary production; and the influence of environmental processes on biofilm formation has been reviewed recently (Battin et al., 2016; Wagner and Lambert, 2018). The formation of a biofilm leads to several advantages for single planktonic cells. Amongst others, the biofilm matrix enables bacterial cells to perform cell–cell interactions/communication and exchange of DNA; the matrix stores nutrients and can act as a barrier against desiccation and serves as a defense mechanism against predation.

Biofilms are part of the aquatic food web and were characterized in a recent review by three major elements: energy pathways/subsidization from plankton, horizontal complexity of the basal food web, and the vertical food web complexity/food chain length (Weitere et al., 2018). Laboratory experiments showed that microplastic particles coated with a biofilm preferentially become ingested by organisms compared with pristine particles (Vroom et al., 2017; Hodgson et al., 2018), which renders biofilm-coated plastic materials a potentially enhanced environmental and organismal health risk (Ramsperger et al., 2020b). Furthermore, bacterial and fungal pathogens have been identified in the plastisphere of freshwater and terrestrial habitats, where microplastic particles can serve as vectors for pathogen distribution (Wu et al., 2019; Gkoutselis et al., 2021).

In the last couple of years, studies showed that emigration, dispersal, and immigration also play a major role for biofilm microorganisms (Augspurger et al., 2010), indicating that differences in the colonization of various plastic types may also account for the composition of microbial communities. Further, microbial communities in natural environments, contrary to laboratory conditions, undergo considerable seasonal dynamics as shown for headwater streams and small rivers in Central Europe (Kaevska et al., 2016) and account for the formation and composition of biofilm microbiomes. However, the few publications from natural freshwater habitats show that only little is known about the colonization and biofilm formation on different polymer types in freshwater ecosystems, which holds even more true for small headwater streams.

To explore the pro- and eukaryotic microbial communities on microplastic particles in a headwater stream, we directly exposed particles of various polymer types for 4 weeks in a small headwater stream in Northern Bavaria, Germany, in two independent experiments (spring and summer) to compensate for seasonal dynamics of freshwater microbiomes. We analyzed the prokaryotic community by 16S amplicon sequencing to elucidate bacterial community differences in colonization and potential degradation of polymer particles. Since prokaryotic biofilms attract eukaryotic predators such as protists and small metazoans, 18S amplicon sequencing was chosen to investigate eukaryotic community dynamics on the biofilms. We investigated whether differences in community functions can be inferred from taxonomic abundances of prokaryotic communities in a seasonal and/or particle-type specific manner. Furthermore, the presence of pathogenic bacteria was analyzed in biofilm and stream water samples.

## Materials and Methods

### Exposition of Plastic Particles in a Freshwater Stream

Plastic particles (approximately 3 mm in diameter) were exposed in the freshwater stream Truppach in northern Bavaria, Germany (lat. 11.36459, lon. 49.89573), about 500 m downstream a local wastewater treatment plant (WWTP) from May 2, 2016, to May 30, 2016 (spring experiment), and from August 13, 2016, to September 9, 2016 (summer experiment), respectively. Water samples of the headwater stream were taken on September 9, 2016, at two locations: the site of the *in situ* exposition experiment and at a site approximately 500 m upstream of the outlet of the WWTP (lat. 11.37370, lon. 49.90627). The water temperature of streams and rivers in this area is monitored by the Bavarian Environment Agency^1^ at a nearby monitoring station at Hollfeld, Bavaria, Germany, and ranged from ca. 11.2 to 13.1°C (spring experiment) and from 13.2 to 13.8°C (summer experiment). The WWTP receives mainly private household wastewater for mechanical and biological (nitrification and denitrification) treatment. The efflux of the WWTP is not sterilized and constituted 3.75% (annual average) of the streamflow in the year of exposition (source: www.lfu.bayern.de and personal communication with the operator of the WWTP). The area next to the sampling site is agriculturally used grassland (partly pasture) and protected by the Flora–Fauna–Habitats directive of the European Commission (Council Directive 92/43/EEC). The properties of the particles used in the exposure experiment are summarized in Table 1. The polymer particles were taken from the original container but were not sterilized prior to the experiment to avoid thermal modification of the particles. The quartz particles were thoroughly rinsed with deionized water to remove quartz dust and were autoclaved. The experimental design was as follows: for each particle type, one basket (ball-shaped stainless-steel sieve, about 4.5 cm in diameter, mesh size ca. 0.65 mm) containing approximately 50 particles was used to expose particles to the stream water, prohibiting contact of particles with floating debris (e.g., branches and litter) as well as access of feeding macrofauna of the stream (e.g., fishes, snails, and insect larvae) to the developing biofilm. The baskets were attached to a wooden raft, held in place by a rope, and always free-floating in the stream at a depth of approximately 20 cm below the water surface without touching the streambed at any time. After the exposure period, the particles were collected separately for each particle type and washed three times with tap water to remove stream water and loosely attached debris from the developing biofilm. Ten particles were randomly taken two times from each particle type pool (unit of replication: 10 particles), transferred to an extraction vial, and kept at 4°C overnight until DNA extraction on the following day. The same experimental design was used for the repetition of the experiment in August/September 2016. Potential microbial contamination of the tap water (used for the initial washing step) was analyzed by amplification of 16S fragments and high-resolution capillary electrophoresis. No 16S amplification products could be detected in any tap water samples, while stream water samples showed specific 16S amplification products (data not shown).

**TABLE 1:** Characteristics of particles used in the experiments.

**Particle type**	**Trade name**	**Article no.**	**Supplier**	**Diameter (mm)**
Low-density polyethylene (PE)	Lupolen	6031M	Pro-Plast Kunststoff GmbH, Weiterstadt, Germany	3
Polypropylene (PP)	Moplen	HP570M	Pro-Plast Kunststoff GmbH, Weiterstadt, Germany	3
Polystyrene (PS)	–	158K KG2	BASF SE, Ludwigshafen, Germany	3
Polyvinyl chloride (PVC)	Troilit	VB537-HE	Granulat GmbH, Troisdorf, Germany	3
Quartz-based gravel (Q, reference particles)	Color Gravel Super White	50260	Colorstone, Rudolstadt, Germany	2–3

### DNA Extraction From Biofilm and Water Samples

Metagenomic DNA of the biofilms was extracted independently (representing technical replicates) using the “PowerBiofilm DNA Isolation Kit” (MO-BIO^2^) as recommended by the manufacturer. In addition, metagenomic DNA was extracted from water samples by ethanol precipitation (Ficetola et al., 2008). The amount of DNA was quantified using the dsDNA High-Sensitivity Assay Kit on a Qubit 3 fluorometer (Fisher Scientific^3^).

### High-Throughput Sequencing of 16S- and 18S-rDNA Amplification Products

The replicate samples of metagenomic DNA were sent to LGC Genomics GmbH^4^ for library preparation and high-throughput sequencing. Briefly, 16S-rDNA fragments were amplified using primer pair Bakt_341F and modified Bakt_805R (GACTACHVGGGTATCTAAKCC, after Herlemann et al., 2011), and 18S-rDNA fragments were amplified using primer pair TAReuk454FWD1 and modified TAReukREV3 (Stoeck et al., 2010; Piredda et al., 2017). PCR amplicons were sequenced in the 300-bp paired-end mode using Illumina’s MiSeq V3 chemistry and instrument. Raw sequence reads were demultiplexed, adapter remnants were clipped from all reads, and forward and reverse primers were removed from the sequences. The sequences were deposited in National Center for Biotechnology Information’s (NCBI’s) Sequence Read Archive (SRA) under project number PRJNA680706.

### Bioinformatics and Statistical Analyses

The microbial composition of the samples was analyzed using the Qiime2 package; a description of the applied workflow is encoded in the provenance tab of Qiime2 Supplementary Data File (a short description on how to view Qiime2 data files is given in a Qiime2 instruction file in Supplementary Material). Briefly, the primer-clipped reads were loaded into the Qiime2 pipeline. The paired-end reads were quality-filtered (including a 3′-end trimming at position 250, where the average read qualities fell below the quality score of Q25 for 16S reads, and Q30 for 18S reads), denoised, and joined by DADA2 (Callahan et al., 2016), yielding so-called amplicon sequence variants (ASVs) (Callahan et al., 2017). Low-abundant ASVs were filtered out based on the median frequency per ASV of 16 (bacterial amplicons) and 17 (18S amplicons). The remaining ASVs were taxonomically classified using “naïve-Bayes” trained taxonomic classifiers based on the SILVA reference database (version 138), containing 16S and 18S reference sequences. Throughout this article, we adhere to the taxonomic nomenclature of the GTDB (prokaryotes) and UniEuk (eukaryotes), which has been adopted with the SILVA v138 reference database release.^5^ Phylogenetic trees were constructed by sequence alignments of 16S and 18S ASVs using MAFFT, followed by masking phylogenetically uninformative or ambiguously aligned columns; FastTree was used to infer phylogenetic trees, which were subsequently rooted at their midpoints. Alpha diversity analyses were performed using core-metrics-phylogenetic workflow of Qiime2, producing several alpha diversity measures (Faith’s PD, evenness, observed ASVs, and Shannon). Beta diversity analyses between sample groups were calculated by DEICODE (Martino et al., 2019) and visualized by QURRO (Fedarko et al., 2020). Statistical differences between sample groups were calculated by ANOSIM and PERMANOVA implemented in Qiime2. Alpha rarefaction curves were generated from the frequency information of the representative ASVs in each sample. The functional prediction tool FAPROTAX (Louca et al., 2016) was used to predict metagenome function from 16S marker gene sequencing profiles. Therefore, taxonomic features obtained by the Qiime2 pipeline were collapsed at the species level. The resulting frequency table was used to predict functional profiles in biofilm, and water samples from the FAPROTAX database provided together with a python script.^6^ Pathogenic bacteria were identified from 16S amplicon reads using the 16sPIP tool (Miao et al., 2017). The 16sPIP package was obtained from the GitHub repository^7^ and run in the sensitive mode with the pair of forward and reverse reads in fastq format for each sample.

## Results

### Amplicon Sequencing Data Overview

Amplicon sequencing of 20 biofilm and 4 water samples obtained from the experiments in spring and summer 2016 resulted in 1.95 million (16S) and 2.94 million (18S) paired-end reads, respectively. The DADA2 plugin (Qiime2) was used to denoise and merge paired-end reads, detect putative chimera, and identify representative ASVs. After rare ASVs were filtered, a final number of 2,521 (16S) and 3,190 (18S) representative ASVs were obtained and used for downstream analyses. Rarefaction analysis of the denoised, chimera-, and quality-filtered reads showed that the remaining sequences were enough to reach saturation for each sample. For alpha and beta diversity analyses of biofilm communities, data corresponding to stream water samples were filtered out from the full dataset.

### Taxonomic Classification of Bacterial and Eukaryotic Amplicon Sequence Variants

**FIGURE 1:**
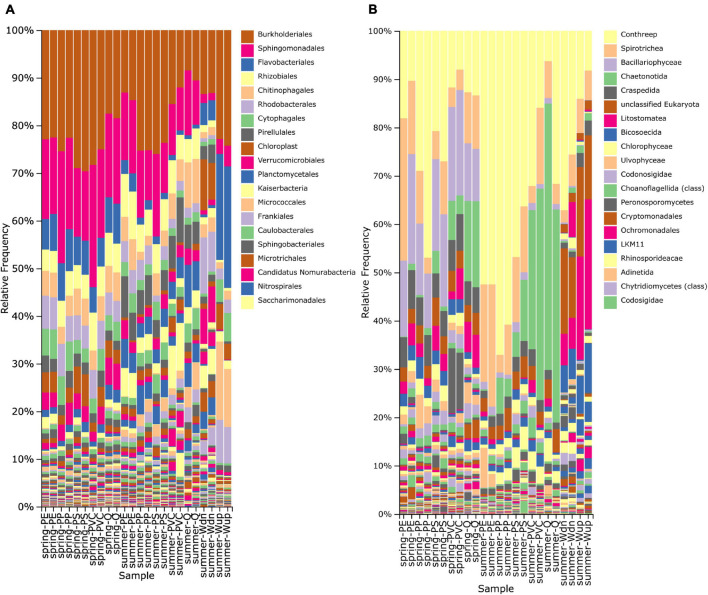
Taxonomy bar plot displaying the relative frequency (%) of prokaryotic **(A)** and eukaryotic **(B)** orders based on taxonomic classification of 16S and 18S rRNA gene fragments. Only the 20 most frequent orders are shown in the legend and are arranged from the most frequent (top) to less frequent taxa (down). The higher taxonomic level is used when the order level is not annotated in the reference database. Biofilm samples are indicated on the horizontal axis as “season-particle” next to water samples taken from the *in situ* exposition site (Wdn) and upstream of the WWTP outlet (Wup).

Representative ASVs were classified using a naïve-Bayes classifier trained on the SILVA 138 reference sequence database (Bokulich et al., 2018; Robeson et al., 2020). The relative frequency of prokaryotic and eukaryotic taxa of biofilm samples differed from microbial communities of the stream water (Figure 1 and Qiime2 taxa bar plots in Supplementary Material). The 10 most abundant orders of bacteria (based on global abundance across all samples) were Burkholderiales, Sphingomonadales, Rhizobiales, Flavobacteriales, Chitinophagales, Rhodobacterales, Pirellulales, Cytophagales, Verrucomicrobiales, and plant chloroplasts (mainly from algae), which contributed to about 40–80% of relative abundance of each sample (Figure 1A).

The number of observed bacterial taxa in biofilm samples ranged from ca. 325 to 583 taxa per particle type and did not differ considerably between May and August samples. PE particles always showed the most detectable bacterial taxa (Table 2A). The evenness of the bacterial microbiome was above 0.9 (Pielou’s index) for most of the samples, indicating that no dominant bacterial group was present (Table 2A). Shannon’s biodiversity index ranged from ca. 7.65 to 8.38 for bacterial taxa, and no remarkable differences were observed between spring and summer samples.

**TABLE 2:** Alpha-biodiversity metrics of (A) pro- and (B) eukaryotic biofilm microbiomes for the spring and summer experiments (mean of replicate samples).

**Particle type**	**Pielou**			**ASV**			**Shannon**		
	**Spring**	**Summer**	**Statistics**	**Spring**	**Summer**	**Statistics**	**Spring**	**Summer**	**Statistics**
**(A) Prokaryota**									
PE	0.912	0.916	a	583	558	a	8.38	8.36	a
PP	0.907	0.925	a	507	438	b	8.14	8.06	a,b
PS	0.925	0.929	a	449	332	b	8.14	7.65	B
PVC	0.897	0.901	b	462	431	b	7.94	7.86	b
Q	0.924	0.930	a	509	325	b	8.31	7.52	a,b
Statistics	a	a		a	a		a	a	
**(B) Eukaryota**									
PE	0.747	0.697	a	557	289	a	6.82	5.70	a
PP	0.696	0.719	a	718	354	a	6.60	6.07	a
PS	0.770	0.725	a	635	459	a	7.14	6.22	a
PVC	0.757	0.638	a	522	286	a	6.83	5.20	a
Q	0.706	0.585	a	700	237	a	6.67	4.61	a
Statistics	a	b		a	b		a	b	

*Calculations were performed by the diversity plugin of Qiime2. Statistical test: Kruskal–Wallis (pairwise) between particle types and seasonal groups; groups with significant difference (*p* < 0.05) are indicated with different letters. PE, polyethylene; PP, polypropylene; PS, polystyrene; PVC, polyvinyl chloride; Q, quartz-based gravel.*

The number of observed eukaryotic taxa ranged from 237 to 718 taxa in biofilm samples, with a clear tendency for more detectable taxa in spring samples (Table 2B). The 10 most abundant eukaryotic orders were Conthreep, Spirotrichea, Bacillariophyceae, Chaetonotida, Craspedida, unclassified Eukaryota, Litostomatea, Bicosoecida, Chlorophyceae, and Ulvophyceae, which summed up to about 60–90% of relative abundance on the particles (Figure 1B). Pielou’s evenness and Shannon’s biodiversity indices were lower for the eukaryotic samples compared with bacterial samples, ranging from ca. 0.585 to 0.770 (evenness) and from 4.61 to 7.14 (Shannon), respectively (Table 2B). Although alpha diversity evenness did not show differences between spring and summer experiments, lower numbers of detectable taxa and lower Shannon’s indices could be seen in the summer samples compared with the spring samples. Alpha rarefaction curves of the eukaryotic dataset showed that the lower number of taxa in the summer samples did not result from insufficient sampling depth as rarefaction curves reached almost saturation even at the sequencing depth of the smallest sample (data not shown).

### Seasonal and Plastic Type-Dependent Differences in Microbial Community Composition

**FIGURE 2:**
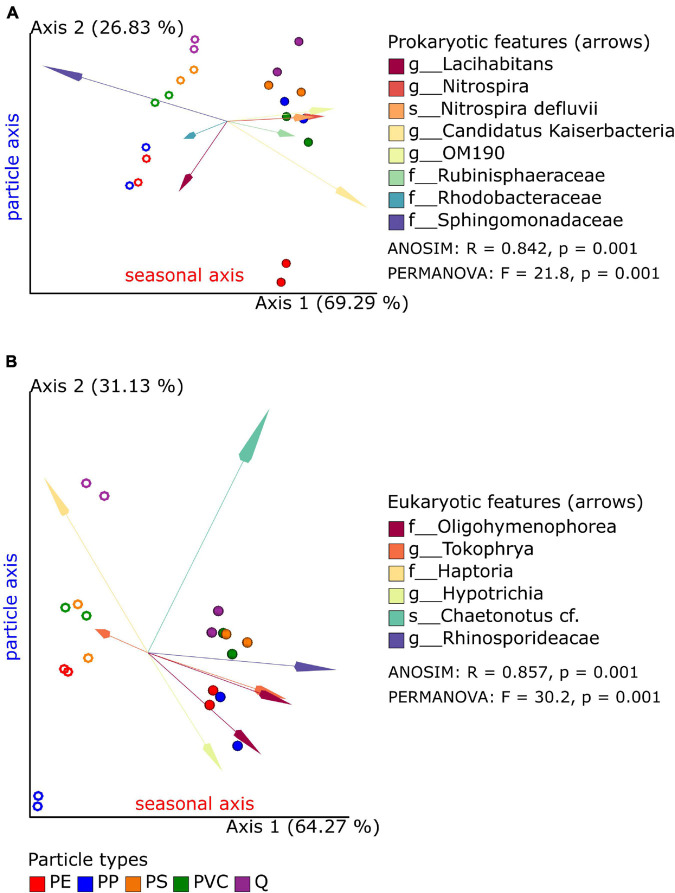
Robust Aitchison principal component analysis (PCA) plot of bacterial **(A)** and eukaryotic **(B)** microbiome samples based on 16S and 18S taxonomic classification and feature (ASV) abundances. Replicate samples of spring (open circles) and summer (closed circles) experiments are displayed together with the most important features (arrows: Euclidian distance from the origin) and their taxonomic classification (merged if several amplicon sequence variants (ASVs) were classified to the same taxon). Results of global ANOSIM and PERMANOVA tests are shown on each plot.

The microbial compositions in biofilms between sample groups (beta diversity) were analyzed by the “Robust Aitchison PCA” (RPCA; implemented in the Qiime2 DEICODE plugin) to address the sparsity and compositional nature of the 16S and 18S datasets (Martino et al., 2019). First, bacterial and eukaryotic microbial communities of the spring and summer exposition experiments exhibit statistically significant separation along RPCA axis 1 (explaining 69 and 64% of the data) in the 16S and 18S dataset (Table 3); the overall direction of community shifts was very similar for all polymer and reference particle types (Figure 2; Qiime2 RPCA plots in Supplementary Material). Second, pro- and eukaryotic microbial communities of the different particle types showed an additional separation along RPCA axis 2, perpendicular to seasonal axis 1 (explaining ca. 26 and 31% of the 16S and 18S data, respectively; Figure 2; Qiime2 RPCA plots in Supplementary Material). Pairwise differences between some particle groups were statistically significant, while other groups overlapped with each other (Table 3).

**TABLE 3:** Results of pairwise statistical tests of sample groups from 16S and 18S datasets.

**Groups**	**PERMANOVA**		**ANOSIM**	
	***p*-Value**	**Pseudo-*F***	***p*-Value**	**Rho**
**16S (prokaryotes)**				
Spring–summer	0.001	14.9	0.001	0.634
PE–PS	0.032	5.47	0.027	0.594
PE–Q	0.024	8.1	0.04	0.718
**18S (eukaryotes)**				
Spring–summer	0.001	16.1	0.001	0.737
PE–Q	*0.055*	*3.82*	*0.067*	*0.427*
PP–PVC	*0.06*	*3.15*	*0.063*	*0.354*
PP–Q	0.028	6.83	0.03	0.667

*Pairwise tests of spring–summer groups included all particle types (*n* = 10), and pairwise tests between particle types included both seasons (*n* = 4). Test results, close to but not reaching the “*p* < 0.05” criteria, are shown in italics. PE, polyethylene; PP, polypropylene; PS, polystyrene; PVC, polyvinyl chloride; Q, quartz-based gravel.*

A common observation was that reference samples were always found on one side of axis 2 in both seasons and the 16S and 18S datasets. Significant differences to quartz samples were found for PE (in 16S data) and PP samples (in 18S data). Although the significance level of 0.05 has not been reached for the PE–Q pair in 18S datasets, the observed *p*-values close to this threshold indicate a putative separation also between these biofilm communities. PS and PVC samples clustered next to each other and in close vicinity to reference samples in the 16S dataset and, therefore, do not show significant differences. This was also true for the 18S dataset in summer experiments, but not for the spring data: here, the PS and PVC samples cluster in the same range of axis 2, while the reference samples are placed apart from these two polymer samples. Finally, pairwise differences within polymer samples could be also observed in the 16S dataset (PE–PS, statistically significant) and in the 18S dataset (PP–PVC, tendency with *p* = 0.060 and *p* = 0.063), respectively. Since axes 1 and 2 together explain over 95% of the data in 16S and 18S RPCA plots, respectively, season and particle types are the most determining factors for microbial diversity in biofilm samples.

### Seasonal Effects on Bacterial and Eukaryotic Microbiomes

A key benefit of RPCA is that sample and feature loadings can be accessed by the QURRO plugin to identify those ASVs at the ends of ordinations, which are the most contributing to the clustering of samples along RPCA axes. Spring and summer samples could be separated along axis 1 in RPCA plots in 16S and 18S datasets. A selection of each 1% from the top and the bottom of ranked features (ASVs) was chosen to retrieve lists of bacterial and eukaryotic taxa, respectively (Supplementary Tables 1A, 2A and Supplementary Figure 1), which differed significantly between spring and summer samples (Supplementary Tables 1B,2B).

In spring biofilms, most of these top-/bottom-ranked bacterial ASVs were classified as Sphingomonadales and Burkholderiales, in addition to Armatimonadales, Chitinophagales, Flavobacteriales, and Verrucomicrobiales. Furthermore, 16S sequences from plant chloroplasts were also abundant in spring samples (Supplementary Table 1A). In summer biofilms, ASVs were classified to Oligoflexales, Nitrospirales, Pirellulales, Planctomycetales, Sphingomonadales, Burkholderiales (different ASVs as the above ones), Steroidobacterales, and Verrucomicrobiales, as well as Candidatus Kaiserbacteria and Candidatus Nomurabacteria; and two clone references were more abundant.

A comparable selection of the most contributing features (1% top/bottom) to RPCA axis 1 of the eukaryotic dataset showed that spring and summer sample groups could be distinguished. The largest eukaryotic group characterizing the spring samples belonged to the phylum Diatomea, e.g., Bacillariophyceae. Furthermore, the phyla Ciliophora, Holozoa, Chlorophyta, and Bicosoecida were identified with two or more ASVs, next to phyla represented by single ASVs (Supplementary Table 2A). Abundant taxa in summer samples belonged mainly to Ciliophora (16 of 28 ASVs), particularly to the two families Oligohymenophorea and Phyllopharyngea, and the phyla Cryptomycota (LKM11), Gastrotricha, and Holozoa.

It should be noted that other thresholds can be applied to the datasets at the reader’s choice using the Qiime2 QURRO visualization files provided as electronic supplements.

### Identification of Polymer-Specific Taxa Within Bacterial and Eukaryotic Biofilms

Particle type-dependent differences in microbial communities occurred mainly along RPCA axis 2, and significant differences between quartz and some polymer-specific communities have been shown above (Table 3). Taxonomic groups responsible for the positioning of each sample along axis 2 of the RPCA plots were identified by QURRO in the same way as described above for seasonal differences along axis 1 in prokaryotic and eukaryotic datasets. The most contributing taxonomic groups were filtered from ranked features (ASVs) of the complete prokaryotic and eukaryotic datasets (using a 3% top/bottom threshold) but also from pairwise datasets composed of each of the polymer types and the reference samples (Q-PE, Q-PP, Q-PS, and Q-PVC; using a 2% top/bottom threshold, accounting for the slightly lower numbers of ASVs present in pairwise subsets). The resulting prokaryotic and eukaryotic taxonomic groups were combined in non-redundant tables, and the association of features (ASVs) to the polymer or the reference sample end of axis 2 was color-coded in these tabular overviews (Supplementary Tables 3A, 4A). Pairwise *t*-tests using the current log-ratio values corresponding to the most contributing taxa between all particle groups confirmed significant differences (and indicated tendencies at slightly higher *p*-values) between those groups, which have been already detected permutation tests on the full 16S and 18S datasets but also revealed considerable overlap between other groups (Supplementary Tables 3B,4B).

### Bacterial Taxa on Microplastic Particles

The list of bacterial taxa contributing to axis 2 of the full and the pairwise datasets consisted of 207 non-redundant ASVs. Thus, a considerable overlap of 47 ASVs could be identified that were present in full and in any of the four pairwise datasets (Supplementary Table 3A). Furthermore, log-ratio plots calculated from selected taxa showed that the sample groups (particle, season) differed considerably (Supplementary Figure 2A): PE samples always showed more negative log-ratio values than other particles, indicating that the denominator taxa were more abundant as compared with the numerator taxa. However, it should be noted that the reference samples (as the most contrasting samples) received log-ratio values in the range from −0.5 to 0, which means that numerator and denominator taxa were present in comparable amounts in these samples (based on RPCA). Interestingly, the log-ratio values of particle groups showed a shift to more negative values in summer samples, which can be caused by either a decrease of the numerator taxa or an increase in the denominator taxa used for log-ratio calculations.

Analysis of pairwise subsets of bacterial data showed a similar trend based on unique selections of taxa from each subset: each particular selection of taxa resulted in a clear separation of microplastic biofilms from the mineral reference biofilms in the spring and summer experiments (for details of pairwise analyses, see QURRO visualizations of pairwise prokaryotic datasets in Supplementary Material).

The non-redundant list of the selected taxa (207 ASVs) is assigned to the orders Chitinophagales, Cytophagales, Flavobacteriales, Candidatus Kaiserbacteria, Pirellulales, Planctomycetales, Rhizobiales, Rhodobacterales, Sphingomonadales, Burkholderiales, and Verrucomicrobiales (containing at least 5 ASVs) and 48 orders with less than 5 ASVs (Supplementary Table 3A). Within Chitinophagales, ASVs classified as Chitinophagaceae (family) were exclusively found in the numerator group (reference particle end). In contrast, some of the Saprospiraceae ASVs were clustering with reference particles and others with polymer particles. Ten ASVs classified to the order of Pirellulales were found exclusively at the polymer particle end of feature rankings. Only one ASV of this order was found within the reference particle end. ASVs of the family Comamonadaceae (order Burkholderiales) represent another taxa example filtered out mainly from the polymer particle end of feature rankings (16 ASVs). Only two belong to the reference particle end.

### Eukaryotic Taxa on Microplastic Particles

The list of eukaryotic taxa extracted from axis 2 of the full and the pairwise RPCA ordination (same percentage filtering as for bacterial data) resulted in 226 non-redundant ASVs with complete overlap of 90 ASVs (Supplementary Table 4A). Log-ratio plots of the selected taxa from the entire dataset showed that the “denominator” taxa are more abundant in PP biofilms (spring and summer) and PE biofilms (summer) in comparison with the “nominator” taxa (Supplementary Figure 2B). Quartz (Q) samples differed most from PP samples with log-ratio values above 3 in spring samples and ca. 0.8 in summer samples. As previously observed for bacterial microbiomes, a shift to more negative log-ratio values could also be observed in the summer experiments for eukaryotic samples.

Log-ratio calculations using taxa obtained from pairwise analyses showed clear separation of most polymer samples from reference samples within each season, except for Q and PVC samples in the summer experiment (see QURRO visualizations of pairwise prokaryotic datasets in Supplementary Material).

The largest taxonomic groups found within the selected taxa (whole and pairwise datasets) were ciliates, e.g., the Conthreep clade, and the orders Litostomatea and Spirotrichea (Supplementary Table 4A). Within the Conthreep clade, ASVs of the family Oligohymenophorea were predominately associated with polymer samples, while ciliates of the families Phyllopharyngea, Prostomatea, Haptoria, and Hypotrichia cluster more with reference samples as well as the family Heterotrichea, another ciliate group assigned to the class Postciliodesmatophora. Several ASVs assigned to green algae (unfortunately with incomplete taxonomic classification from order to genus levels in the SILVA reference database) were also enriched in polymer biofilms (e.g., *Chaetophora incrassata*, *Microspora* sp., *Oedocladium prescottii*, and *Radiococcus* sp. in addition to two ASVs with lacking classification). Several ASVs of the phylum Peronosporomycetes were identified from Q-PVC datasets, indicative of PVC samples. However, the available taxonomic classification is incomplete in SILVA; this group was previously called Oomycota and belongs to the SAR clade of primarily unicellular eukaryotes. A few other groups were enriched in reference samples, such as Nematodes of the order Diplogasterida and Monhysterida, except for one ASV of the order Chromadoria was identified in full and all four pairwise datasets as indicative for polymer particles. A large group of nine ASVs was classified as Rotifera, multicellular animals present in almost all habitats, six of them from the reference particle end of feature rankings, two from PS/PVC specific rankings, and one each from Q (whole dataset) and PVC (Q-PVC dataset) ranking (Supplementary Table 4A).

### Functions of Bacterial Communities

**FIGURE 3:**
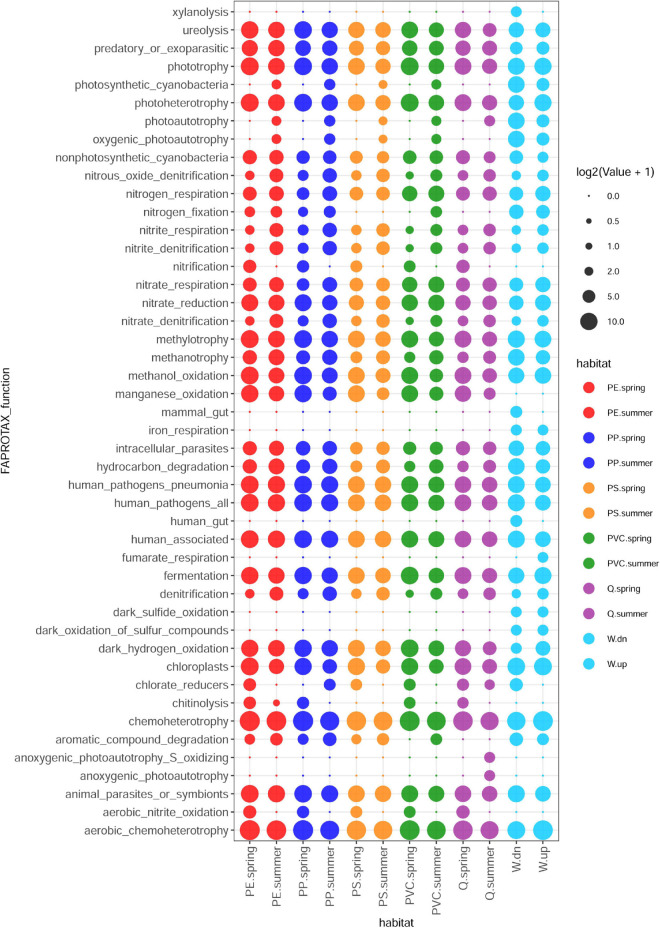
Prediction of metabolic and ecologically relevant functions from 16S taxa abundances by FAPROTAX in different biofilm and planktonic samples.

The functions of bacterial communities were predicted by FAPROTAX (Louca et al., 2016) and revealed a broad range of functional similarities between the biofilm and stream water communities but showed also differences in relative abundance between different polymer, quartz, and water samples, as well as between spring and summer experiments. Functions detected in biofilm, but not in water samples, were aerobic nitrite oxidation, chitinolysis, manganese oxidation, and nitrification. Some functions are more abundant in spring samples within these groups, such as nitrification and aerobic nitrite oxidation. In contrast, other closely related functions such as photosynthetic cyanobacteria, photoautotrophy, and oxygenic photoautotrophy were more abundant in biofilm samples collected in summer. Discriminating functions between polymer and quartz samples are rare, except for aromatic compound degradation and photosynthetic cyanobacteria/oxygenic photoautotrophy, which were not detected on quartz samples. Although many functions detected in stream water communities were also observed in biofilm communities, some functions were prevalent to stream water communities, such as dark oxidation of sulfur compounds, dark sulfite oxidation, and iron respiration, and – more restricted to water samples collected downstream of the WWTP outlet (the site of the exposition experiment) – functions related to xylanolysis, animals, and the human gut, respectively (Figure 3).

### Pathogen Identification in Biofilm Communities

In addition to the taxonomy-based identification of potential human pathogens by FAPROTAX (see above), a sequence-based identification of putative pathogens was performed using the 16sPIP tool and a manually curated pathogen database (Miao et al., 2017). Sixty-nine putative pathogenic bacteria were identified in at least one biofilm or water sample (34 taxa overlapping between biofilm and water samples, 21 taxa only detected in biofilm samples, and 14 taxa only detected in water samples). The most abundant putative pathogen, *Enterobacter ludwigii*, was detected in all samples but exhibited higher relative abundance (up to 0.173%) in biofilm samples than water samples. The seven most abundant pathogens (*E. ludwigii*, *Aeromonas hydrophila*, *Nocardia farcinica*, *Afipia broomeae*, *Pseudomonas aeruginosa*, *Acinetobacter lwoffii*, and *Klebsiella pneumoniae*) represented together over 80% of the sequence reads of putative pathogenic bacteria (Supplementary Figure 3).

## Discussion

Our study investigated whether prokaryotic and eukaryotic biofilm communities differ in their microbial composition between particle types (polymers and quartz) and between two experimental timepoints (seasons) within 1 year. We could show that bacterial and eukaryotic microbiomes cluster between biofilms by only two factors, a seasonal factor and a particle type-specific factor, both together explaining approximately 95% of data variation. Notably, the relative position of biofilm samples along the particle type-specific factor exhibited strong similarities between spring and summer samples, suggesting that substrate characteristics were responsible for biofilm formation in independent experiments.

It has to be noted that the species composition of biofilms is difficult to compare between different studies. Next to the influence of season, the initial species composition may be highly different between ecosystems, or even similar ecosystems may differ in their microbial species compositions due to different biotic and abiotic factors and, thus, influence biofilm formation and composition (see Figure 4 in Battin et al., 2016). Furthermore, *in situ* exposure experiments are hardly comparable with laboratory studies concerning species composition. Microbial species of sampled water represent a one-time inoculum and differ considerably from the dynamic inoculum of streams. The exposition site selected for our experiments is located ca. 500 m downstream of the outlet of a WWTP, which is the first plant along this headwater stream (source: urban wastewater map at www.thru.de). Having these thoughts in mind, we attempt to compare our results with similar studies in the following sections.

The most abundant prokaryotic phyla in biofilm samples were Proteobacteria, Bacteridota, and Planctomycetota, representing up to 80% of the relative abundance in these samples. On the other hand, planktonic water samples differed mainly from biofilm samples by the relatively high abundance of the class Actinobacteria and the lower relative abundance of Planctomycetes. The overall prokaryotic composition of biofilm and planktonic samples in our study largely coincides with results of a recent meta-analysis of plastisphere communities from different environments, including freshwater plastisphere and plankton (Wright et al., 2021).

Applying the high resolution power of RPCA (Martino et al., 2019) on sparse compositional microbiome datasets and filtering ranked ASVs from the ordination ends, we could show in our study that prokaryotic taxa of the Saprospiraceae (Bactegroidia), Pirellulales (Planctomycetota), and Comamonadaceae (Gammaproteobacteria) were detected multiple times (independent ASVs) as enriched taxa on microplastic particles compared with reference particles. Recently, a laboratory study of developing biofilms on PE, PP, and other natural particles incubated in sampled freshwater from Xuanwu lake (China) revealed Gammaproteobacteria as one of the bacterial groups that were more abundant (based on relative frequency) on PE particles compared with other particles (Miao et al., 2019). This observation agrees in part with our findings from exposure experiments performed in a natural environment, as the ASVs of the Comamonadaceae (Gammaproteobacteria) were highly indicative for polymer-specific biofilms. In contrast, other Gammaproteobacteria taxa were more indicative for reference samples.

The most abundant eukaryotic taxa detected in our experiments were Ciliophora, Diatomea, Holozoa, and Gastrotricha, representing ca. 60–85% of relative frequencies in the samples. These findings overlap in part with eukaryotic taxa found on plastic particles in brackish ecosystems (Kettner et al., 2019), despite that freshwater streams and brackish coastal water constitute quite different environments. A potential eukaryotic key taxon for PE biofilms in brackish water has been identified as an unclassified Monogononta (phylum Rotifera) by Kettner et al. (2019). Still, it remains unclear whether the corresponding 18S sequence is similar/identical to any of the Rotifera ASVs identified in our experiments. Furthermore, additional ASVs classified as Rotifera were enriched on reference particles, and only two ASVs (order Ploimidia, class Monogononta) were more abundant on PS and PVC samples. Eukaryotic biofilms on low-density PE (LDPE) membranes have also been investigated by ARISA and sequencing clone libraries in the Marne river (Fechner et al., 2010). Although the sequencing depth is not comparable with 18S metabarcoding, the obtained 5.8S sequences also represented mainly diatoms and ciliates.

In our study, many ASVs assigned to Ciliophora have been identified as accountable taxa to the clustering of samples along the polymer axis of the RPCA plot. This phylum diverged from other eukaryotes approximately 1,143 Ma ago and split into large classes such as CONThreeP (in SILVA: Conthreep) and Spirotrichea (Fernandes and Schrago, 2019). Although ciliates were ubiquitously identified on polymer and reference particles, members of the Oligohymenophorea family (Conthreep clade) were more associated with polymer particles, in particular PE and PP, while members of the families Phyllopharyngea and Prostomatea (Contreep clade), Haptoria (order Litostomatea), and Hypotrichia (order Spirotrichea) were equally distributed or even more often associated with reference particles (Q). An extensive review on the functional diversity of aquatic ciliates (Weisse, 2017) has shown that ciliates cover many ecological niches and a broad range of trophic layers ranging from heterotrophs (bacterivores, herbivores, omnivores, carnivores, etc.) to symbionts (commensals, parasites, mixotrophs, and even photoautotrophs). The differential abundance of distinct ciliate taxa (distinct ASVs) in the different polymer-specific biofilms could possibly be explained by such a ciliate diversification into different ecological niches and shows that a complex biofilm community with different trophic layers has been developed even on a comparably young biofilm of only 4 weeks.

With respect to biofilms on different plastic types, the development and succession of biofilms of different plastic and glass substrates have been investigated in marine environments by scanning electron microscopy and 16S sequencing (Pinto et al., 2019). Significant differences in the bacterial communities were observed on PVC compared with other plastic types and glass surfaces after 10 days of exposure in this natural saltwater environment. This observation agrees well with recent findings that the early-stage biofilm formation on microplastics depends on environmental medium and polymer properties, particularly with the comparably high structural diversity of PVC samples in seawater compared with freshwater (Ramsperger et al., 2020a). If comparable fast colonization also occurred in our freshwater experiments, it is intriguing to assume that slightly different bacterial communities on particles can influence the subsequent colonization of the particles by specified eukaryotic predators. Such trophic predator–prey interactions certainly occur multiple times in natural biofilms and highlight the need to investigate these interactions at the basis of the food web in much more detail to explore the fate and persistence of microplastics in the environment.

Prediction of functional abundances in bacterial communities of different polymer types and the surrounding water samples highlighted functional profiles that differed between water and biofilm samples, between the two seasons, and between various particle types. A possible contribution of the WWTP effluent to microbial communities could be detected in functions ascribed to mammalian gut bacteria and xylanolysis (occurring during digestion of plant material in the rumen), which were only found in water samples at the experiment site (downstream of the WWTP) but not in water samples collected upstream of the WWTP. However, gut-related functions were almost absent in biofilm communities of plastic and quartz particles, indicating that gut-related microbes present in stream water do hardly contribute to biofilm communities. Although the putative origin of gut-affiliated functions from the outflow of the WWTP is intriguing, other potential sources such as cattle grazing areas with surface water runoffs entering the stream upstream of the experiment site but downstream of the second water collection site cannot be ruled out. Furthermore, functions related to photoautotrophy, e.g., cyanobacteria, are more abundant in summer, suggesting an increase in photosynthetic microorganisms in biofilms as described for cyanobacterial bloom in rivers and lakes (Larsen et al., 2020). On the other side, nitrification (including aerobic nitrite oxidation) is more abundant in spring samples as it has been observed in freshwater lakes (Massé et al., 2019). Aerobic ammonia oxidation, another sub-function within nitrification, has not been detected by FAPROTAX in any sample, although included in the FAPROTAX database. Differentiation between organic polymers and inorganic quartz samples is weak and only detected with photosynthetic cyanobacteria/oxygenic photoautotrophy and for aromatic compound degradation. The latter function has also been described as an enriched function in the plastisphere of freshwater and saltwater (Li C. et al., 2021). However, more experiments are necessary to investigate whether metabolic processes of aromatic compounds correlate with organic (and potentially degradable) surfaces (in contrast to quartz particles).

Pathogenic bacteria, identified based on 16S sequence comparison (16sPIP tool), were detected in biofilm and water samples at the same abundances described for sewage and WWTP (Li D. et al., 2021). Few bacterial species dominated the profile of pathogenic bacteria in biofilm samples; the seven most abundant pathogenic bacteria already represented over 80% of the 69 identified pathogenic bacteria in biofilm samples. *E. ludwigii* has been recently identified in a case of catheter-associated bloodstream infection with massive aggregation of the bacterium outside the central venous catheter (Wagner et al., 2020). Other pathogens have also been reported as colonizers of PE and PP (*A. hydrophila*: Thomas et al., 2020), as possible degraders of LDPE and as microplastic surface colonizers (*N. farcinica*: Soleimani et al., 2021; Yi et al., 2021), or were identified as so-called ultramicrocells from drinking water systems made of PVC pipes (*Afipia* sp.: Silbaq, 2009). Identifying pathogenic bacteria at similar percentages over a broad range of polymer and quartz surfaces suggests that these potentially harmful microorganisms can colonize many surfaces.

Our results contribute to the knowledge of microplastic biofilm communities, and it is to our best knowledge the first report of parallel analysis of prokaryotic and eukaryotic (mainly protists) communities on various polymers in headwater streams. We show that polymer-specific community differences can be identified from complex microbiome datasets despite the seasonal dynamics of biofilm communities. While functional differences in biofilm communities could be detected along the seasonal gradient, polymer- and quartz-specific functional differences are weak (but not absent) and should be further investigated in the future. Finally, a surprisingly strong correlation of polymer-specific clustering in prokaryotic and eukaryotic communities could be discovered, which could serve as a starting point for designing new experiments in microplastic research.

## Data Availability Statement

The datasets presented in this study can be found in online repositories. The names of the repository/repositories and accession number(s) can be found below: NCBI’s Sequence Read Archive (SRA), Bioproject PRJNA680706.

## Author Contributions

AW and CL created the project idea. AW conducted the experiment, processed the samples, analyzed that data, and wrote the manuscript. ML, AR, and CL revised the manuscript. All authors contributed to the article and approved the submitted version.

## Conflict of Interest

The authors declare that the research was conducted in the absence of any commercial or financial relationships that could be construed as a potential conflict of interest.

## Publisher’s Note

All claims expressed in this article are solely those of the authors and do not necessarily represent those of their affiliated organizations, or those of the publisher, the editors and the reviewers. Any product that may be evaluated in this article, or claim that may be made by its manufacturer, is not guaranteed or endorsed by the publisher.

## Footnotes

1
www.lfu.bayern.de

2
www.mobio.com

3
www.fishersci.co.uk

4
www.lgcgroup.com

5
https://www.arb-silva.de/documentation/release-1381/

6
http://www.loucalab.com/archive/FAPROTAX/

7
github.com/jjmiao1314/16sPIP
