# Supplementary material for: In situ Prokaryotic and Eukaryotic Communities on Microplastic Particles in a Small Headwater Stream in Germany
Source: Front Microbiol. 2021 Nov 29;12:660024. doi: 10.3389/fmicb.2021.660024 (PMC8667586; doi:10.3389/fmicb.2021.660024)
Supplement: Supplementary file 1 [file Data_Sheet_1.ZIP › Figure S1.pdf]

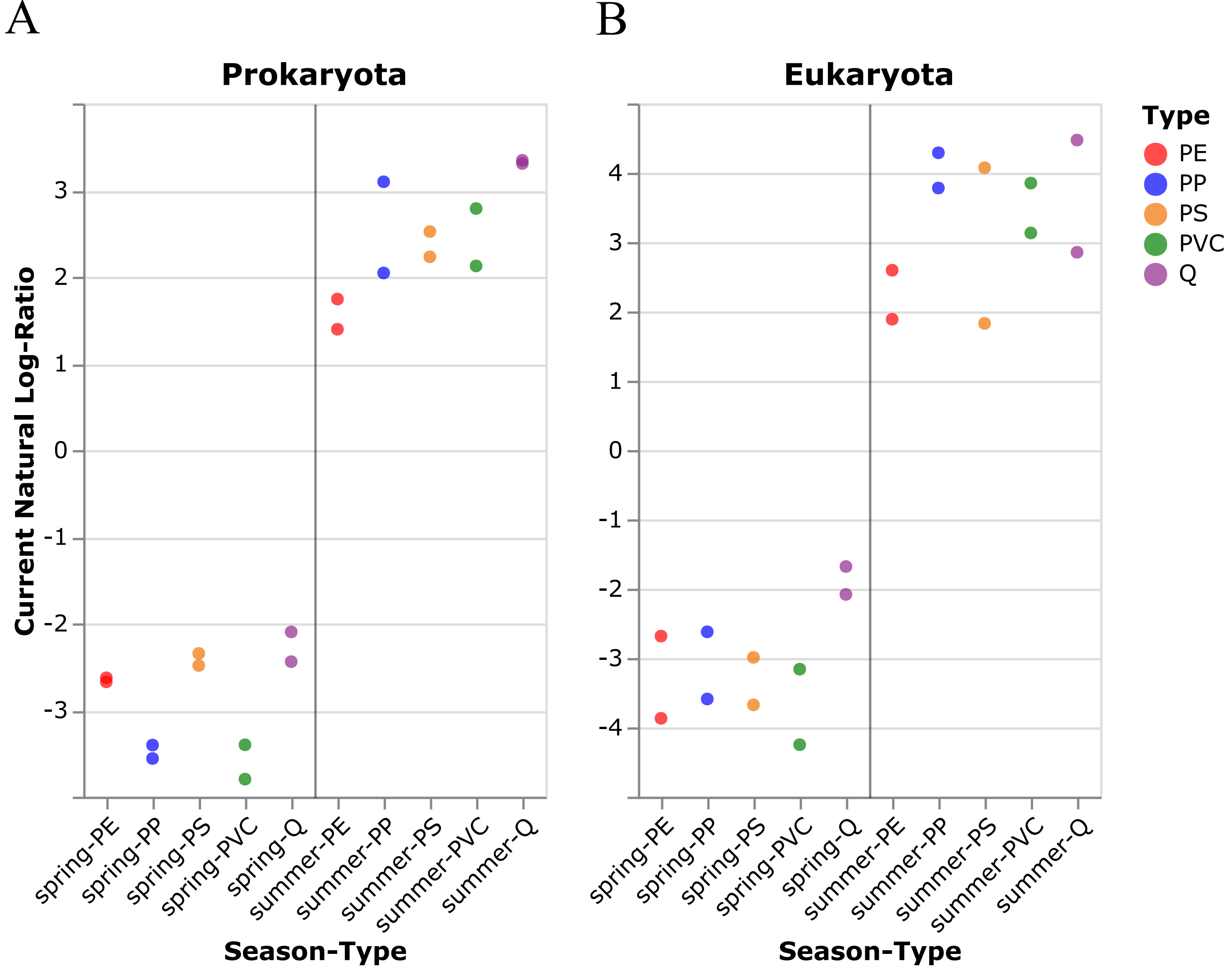

Figure S1: Log-ratio calculations of most-contributing features of prokaryotic (A) and eukaryotic (B) data sets to the season-specific axis 1 in RPCA plots. The respective list of features (ASVs) were filtered from RPCA data files using a 1% autoselection threshold (QURRO plugin to Qiime).
