# Supplementary material for: In situ Prokaryotic and Eukaryotic Communities on Microplastic Particles in a Small Headwater Stream in Germany
Source: Front Microbiol. 2021 Nov 29;12:660024. doi: 10.3389/fmicb.2021.660024 (PMC8667586; doi:10.3389/fmicb.2021.660024)
Supplement: Supplementary file 1 [file Data_Sheet_1.ZIP › Figure S3.pdf]

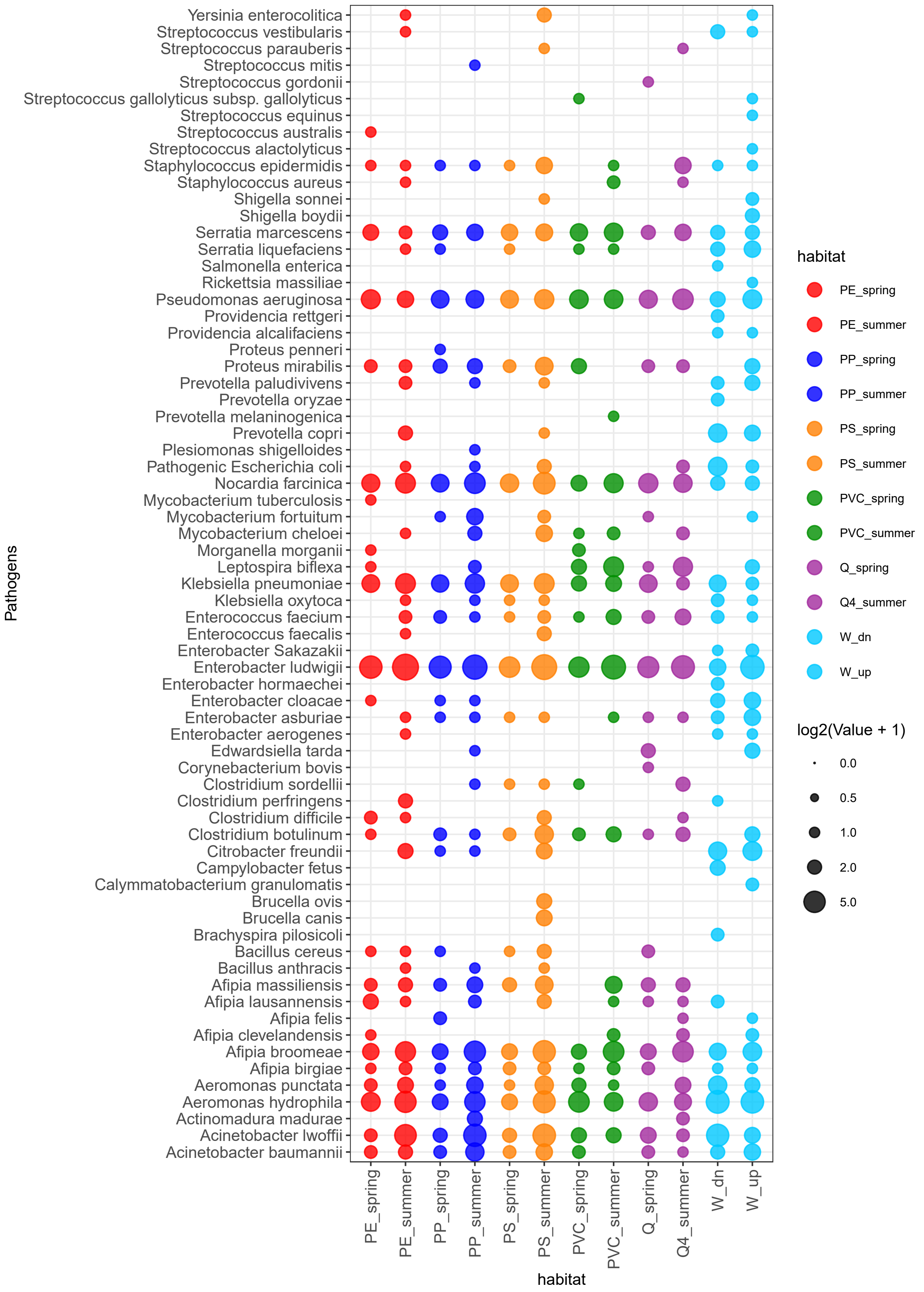

Figure S3: Potential pathogenic bacteria in biofilm and water samples, predicted by 16sPIP (Miao et al., 2017)
