# Supplementary material for: In situ Prokaryotic and Eukaryotic Communities on Microplastic Particles in a Small Headwater Stream in Germany
Source: Front Microbiol. 2021 Nov 29;12:660024. doi: 10.3389/fmicb.2021.660024 (PMC8667586; doi:10.3389/fmicb.2021.660024)

Instructions to open Qiime2 data by Qiime2view Web Interface

Content

1 QIIME2 analysis parameters:..2
2 Qiime2 taxa barplots 3
3 Qiime2 RPCA plots..4
4 Qiime2 QURRO..5

1 QIIME2 analysis parameters:

Any Qiime2 data file contains metadata information how it was generated (provenance). This information is available in the ‚Provenance‘ tab of any Qiime2 data file and can be access via the Qiime2view interface https://view.qiime2.org/.

- The parameters used for each analyses step can be displayed by ‘Action Details’;
- The type / format of each analysis output can be displayed by ‘Result Details’;

Instruction (graphical example below):

1. Open any Qiime2 data file in **Qiime2view (https://view.qiime2.org/)**
2. Select the ‚**Provenance**‘ tab on the top right side
3. All analysis steps leading to the current Qiime2 data file are displayed in a graphical view
    a. optional: select and move single elements for better viewing
4. Metadata information of each analysis step can be displayed by clicking on one of the two graphical elements:
    a. **Rectangle**: Display of **action details**, including **action** and **parameters**.
    b. **Circle**: Display of **result details**, including **type** and **format** of the output data.

Detailed information one Qiime2’ data files are available at https://docs.qiime2.org/ under the section ‘Core concepts’.

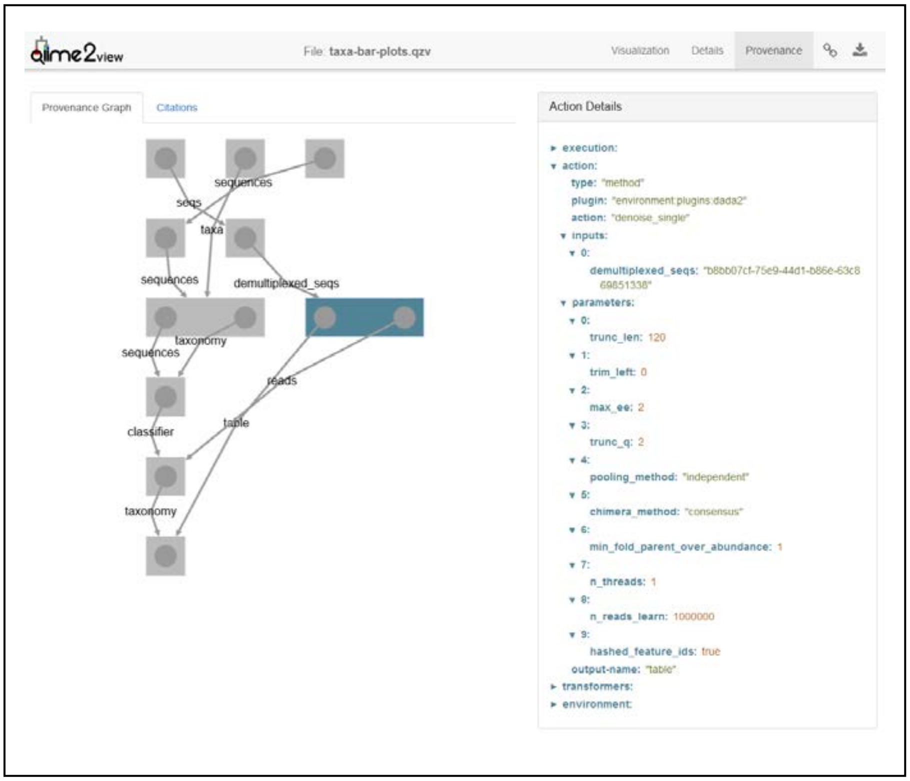

2 Qiime2 taxa barplots

Instruction (graphical example below):

1. Open Qiime2 barplot file in **Qiime2view (https://view.qiime2.org/)**
   a. 16S data: taxa-barplot_Figure-1A_Prokaryotes.qzv
   b. 18S data: taxa-barplot_Figure-1A_Eukaryotes.qzv
2. Choose taxonomic level for display
   a. Level 1 (domain)
   b. …
   c. Level 7 (species)
3. Sort sample on horizontal axis by one or more metadata field; available metadata are:
   a. Season (sping, summer)
   b. Type (particle type)
   c. SeasonType (combined from above)
   d. TypeSeason (combined from abobe)
   e. index (library name)
4. The standard color palette will be used for display;
5. Individual taxonomic groups can be selected by clicking on the color in the legend

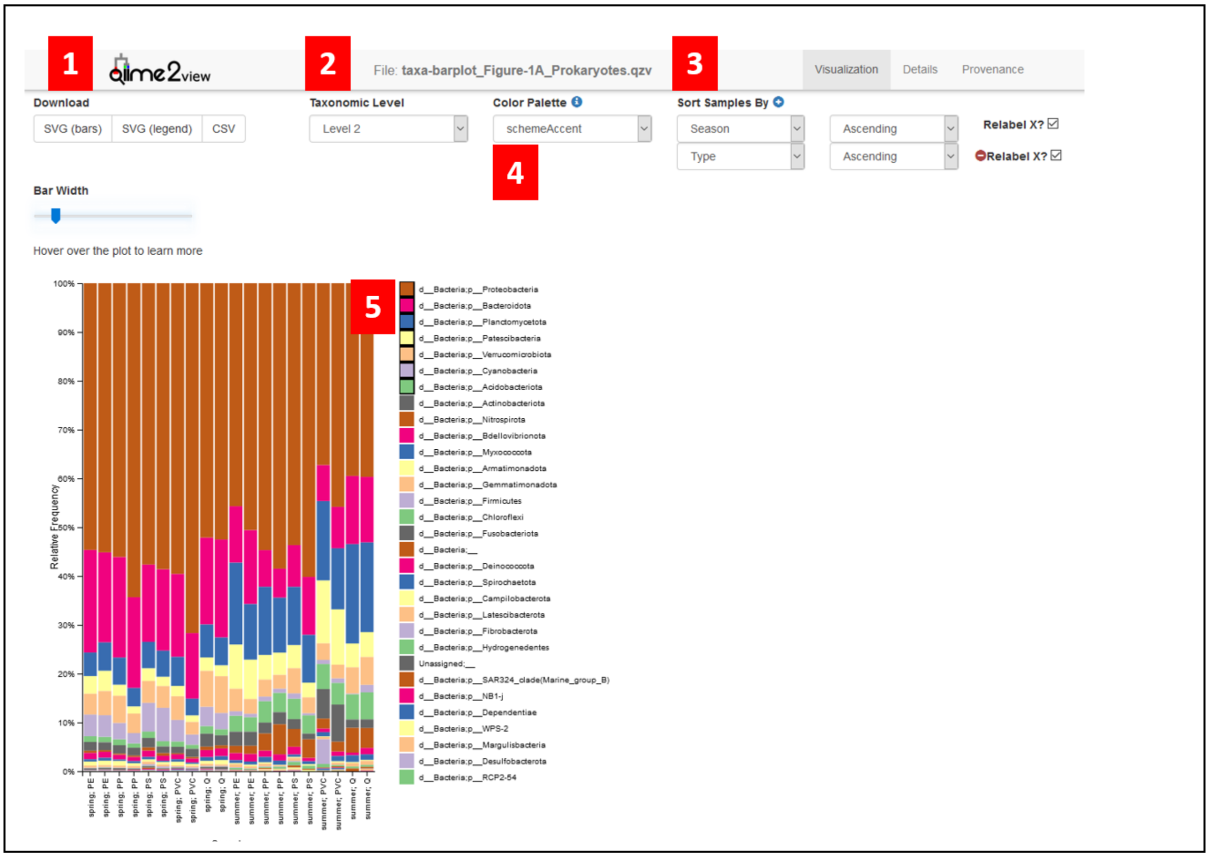

3 Qiime2 RPCA plots

Instructions (graphical example below)

1. Open Qiime2 RPCA (biplot) file in **Qiime2view (https://view.qiime2.org/)**
    a. 16S RPCA plot: biplot_RPCA_Figure-2A_Prokaryotes.qzv
    b. 18S RPCA plot: biplot_RPCA_Figure-2A_Eukaryotes.qzv
2. Color dots and arrows in the plot by **Color > Select a color category**:
    a. Scatter: Type
    b. biplot: Taxon
3. Change dots in the plot by **Shape > Select a shape category**
    a. Scatter: Season, then change ‘spring’ dots to ‘ring’
4. optional: Click in the plot and rotate axes
5. optional: Zoom plot with mouse wheel

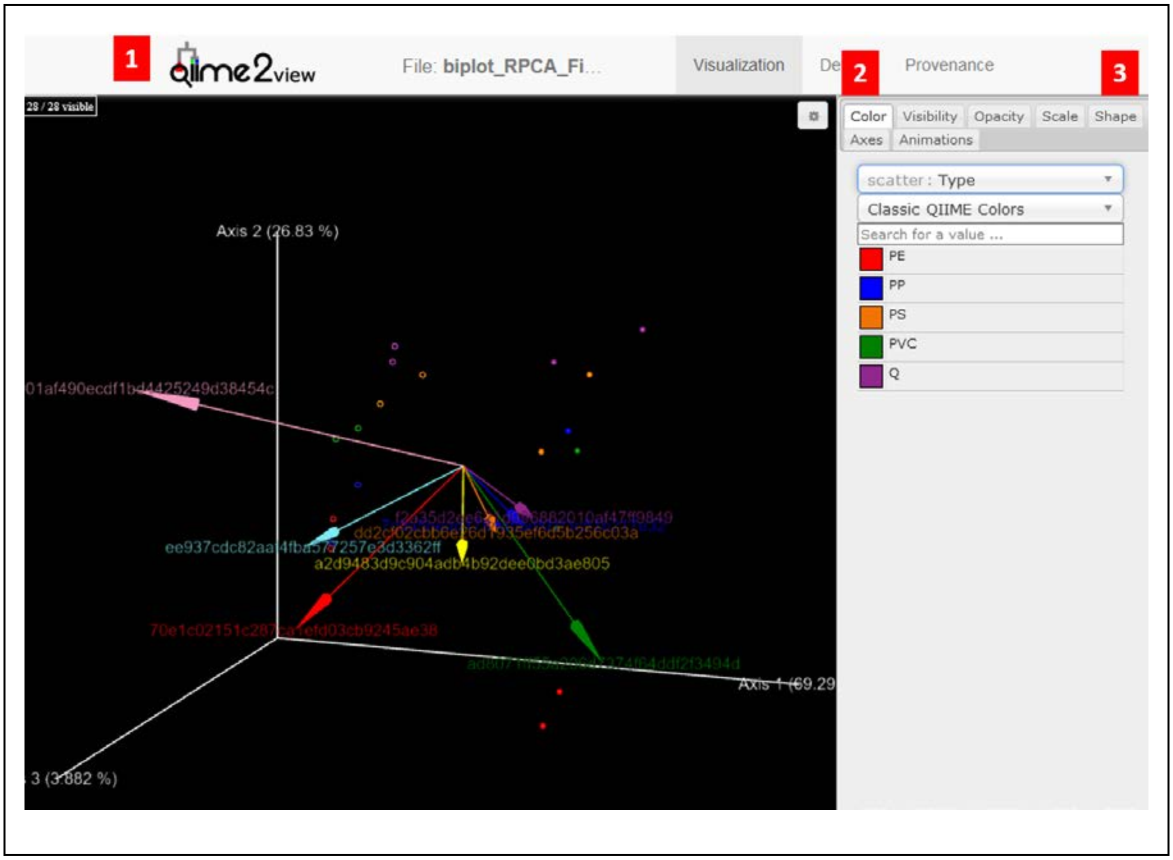

4 Qiime2 QURRO

Feature rankings of RPCA plot can be visualized by QURRO

Instructions (graphical example below)

1. Open Qiime2 qurro loading plot file in **Qiime2view (https://view.qiime2.org/)**
    a. full datasets:
        i. 16S: qurro-plot_full_Prokaryotes.qzv
        ii. 18S: qurro-plot_full_Eukaryotes.qzv
    b. pairwise datasets:
        i. 16S: qurro-plot_Q-PE_Prokaryotes.qzv (and Q-PP, Q-PS, Q-PVC)
        ii. 18S: qurro-plot_Q-PE_Eukaryotes.qzv (and Q-PP, Q-PS, Q-PVC)
2. Select axis for feature loadings
    a. axis 1: season
    b. axis 2: particles
    c. optional: check ‘fit bar width …” to scale the rank plot
3. Autoselect highest/lowest ranked features and confirm by ‘Apply’
    a. see main text for thresholds used Figure S1 and S2
4. Change x-axis field below the sample plot
    a. Season: spring, summer
    b. Type: PE, PP, PS, PVC, Q
    c. SeasonType: spring-PE, spring-PP, …, summer-Q
5. Taxa of the current selection used for Numerator and Denominator in log-ratio calcualtions are displayed in the lower left corner of the Qiime2view window.

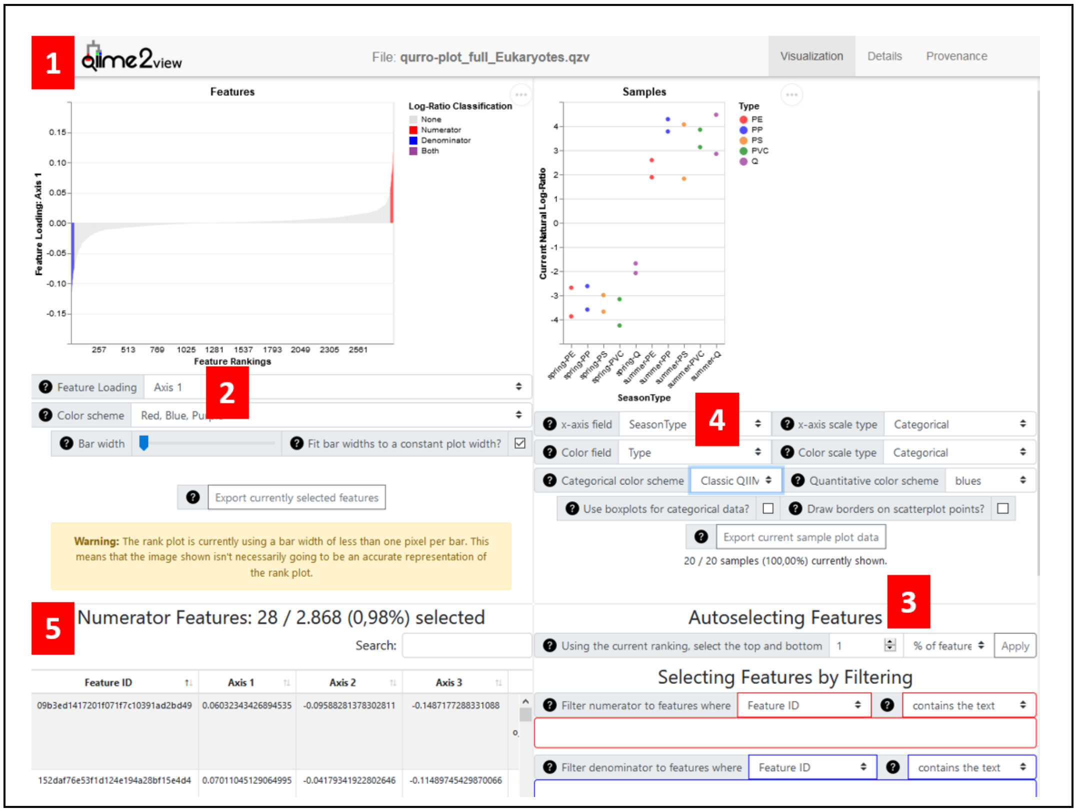

Supplement: Supplementary file 1 [file Data_Sheet_1.ZIP › Intruction_to_use_Qiime2_data.pdf]
