# Supplementary material for: In situ Prokaryotic and Eukaryotic Communities on Microplastic Particles in a Small Headwater Stream in Germany
Source: Front Microbiol. 2021 Nov 29;12:660024. doi: 10.3389/fmicb.2021.660024 (PMC8667586; doi:10.3389/fmicb.2021.660024)
Supplement: Supplementary file 1 [file Data_Sheet_1.ZIP › Supplementary_Material.pdf]

Supplementary Material

1 List of Supplementary Material

1.1 Tables

- Table S1.xlsx: Excel spreadsheets containing the most contributing prokaryotic features along axis 1 (seasonal axis) including t-test results
- Table S2.xlsx: Excel spreadsheets containing the most contributing eukaryotic features along axis 1 (seasonal axis) including t-test results
- Table S3.xlsx: Excel spreadsheets containing the most contributing prokaryotic features along axis 2 (particle axis) including pairwise t-test results
- Table S4.xlsx: Excel spreadsheets containing the most contributing eukaryotic features along axis 2 (particle axis) including pairwise t-test results

1.2 Figures

- Figure S1.pdf: Current log-ratio plots for RPCA axis 1 (seasonal axis) for prokaryotic and eukaryotic data sets
- Figure S2.pdf: Current log-ratio plots for RPCA axis 2 (particle axis) for prokaryotic and eukaryotic data sets
- Figure S3.pdf: Pathogenic bacteria detected in biofilm and water samples

1.3 Qiime2 Information and Data Files

- Intruction_to_use_Qiime2_data.pdf: Instructions to open Qiime2 data by Qiime2view Web Interface (view.qiime2.org), including instructions how to access Qiime2 analysis parameters via the provenance tab;
- Taxonomic barplots:
    - taxa-barplot_Figure-1A_Prokaryotes2.qzv
    - taxa-barplot_Figure-1B_Eukaryotes2.qzv
- RPCA biplots
    - biplot_RPCA_Figure-2A_Prokaryotes.qzv
    - biplot_RPCA_Figure-2B_Eukaryotes.qzv
- QURRO plots
    - qurro-plot_full_Prokaryotes.qzv
    - qurro-plot_full_Eukaryotes.qzv
    - qurro-plot_Q-PE_Eukaryotes.qzv
    - qurro-plot_Q-PE_Prokaryotes.qzv
    - qurro-plot_Q-PP_Eukaryotes.qzv
    - qurro-plot_Q-PP_Prokaryotes.qzv
    - qurro-plot_Q-PS_Eukaryotes.qzv
    - qurro-plot_Q-PS_Prokaryotes.qzv
    - qurro-plot_Q-PVC_Eukaryotes.qzv
    - qurro-plot_Q-PVC_Prokaryotes.qzv
